# Characterizing Adsorption Performance of Granular Activated Carbon with Permittivity

**DOI:** 10.3390/ma10030269

**Published:** 2017-03-07

**Authors:** Yang Yang, Chao Shi, Yi Zhang, Jinghua Ye, Huacheng Zhu, Kama Huang

**Affiliations:** College of Electronic and Information Engineering, Sichuan University, Chengdu 610065, China; yyang@scu.edu.cn (Y.Y.); shichaoscu@126.com (C.S.); yizhangscu@126.com (Y.Z.); 2015322050010@stu.scu.edu.cn (J.Y.); kmhuang126@126.com (K.H.)

**Keywords:** permittivity, pore volume, granular activated carbon

## Abstract

A number of studies have achieved the consensus that microwave thermal technology can regenerate the granular activated carbon (GAC) more efficiently and energy-conservatively than other technologies. In particular, in the microwave heating industry, permittivity is a crucial parameter. This paper developed two equivalent models to establish the relationship between effective complex permittivity and pore volume of the GAC. It is generally based on Maxwell-Garnett approximation (MGA) theory. With two different assumptions in the model, two quantificational expressions were derived, respectively. Permittivity measurements and Brunauer–Emmett–Teller (BET) testing had been introduced in the experiments. Results confirmed the two expressions, which were extremely similar. Theoretical and experimental graphs were matched. This paper set up a bridge which links effective complex permittivity and pore volume of the GAC. Furthermore, it provides a potential and convenient method for the rapid assisted characterization of the GAC in its adsorption performance.

## 1. Introduction

Granular activated carbon (GAC) has a remarkable adsorption capacity due to its extensively developed internal pore structure and large specific surface area [1], making it an ideal medium for adsorbing impurities and purifying both aqueous solutions [2,3,4] and flue gas [5]. To recycle and conserve the limited resources, the GAC needs to be regenerated. Microwave thermal treatment for regenerating GAC is promising [6,7,8]. For instance, Ania et al. [9] studied the effect of different heating mechanisms (a conventional electric furnace versus microwave device) to regenerate the activated carbon exhausted with phenol. Regeneration time was considerably shortened in the microwave device compared to regeneration with the conventional electric furnace. Meanwhile, the porous structure of the regenerated AC using the microwave device was more efficient than that using the conventional electric furnace.

However, the conventional characterization method to evaluate the adsorption performance of the generated GAC is time-consuming. It usually takes dozens of hours to accomplish the test [10]. This paper established the relationship between effective permittivity and pore volume of the GAC. Many studies indicate that either pore volume [11] or pore structure [12], especially pore size distribution (PSD) [13,14] is the most important adsorbent property for GAC. With the development of the microwave measurement method in complex permittivity, e.g., resonant cavity method [15,16,17], perturbation method [15,18,19], transmission and reflection method [20,21,22], it is convenient to get the complex permittivity data of the GAC.

This paper proposes two equivalent models to establish the relationship between effective complex permittivity (ε*_eff_*) and pore volume (*v_g_*) for a given commercial GAC. Brunauer–Emmett–Teller (BET) testing and microwave permittivity measurement with perturbation method are introduced below to verify the relationship between effective complex permittivity and pore volume.

## 2. Derivation

The Maxwell-Garnett (MG) effective medium theory [23], originally derived by neglecting the density fluctuations of dipolar character of scatterers, is the most widely used theory to characterize the complex permittivity of medium [24]. Li et al. [25] applied it in characterizing complex permittivity of solids. It can predict the effective permittivity of a mixture based on the equation:

(1) $$\left(\frac{\varepsilon_{m}-\varepsilon_{h}}{\varepsilon_{m}+2\varepsilon_{h}}\right)=\delta_{i}\left(\frac{\varepsilon_{i}-\varepsilon_{h}}{\varepsilon_{i}+2\varepsilon_{h}}\right)$$

where ε*_m_*, ε*_h_* and ε*_i_* are the complex permittivity of the mixture, host medium and inclusions, respectively, and δ*_i_* is the volume fraction of the inclusions. Equation (1) is valid under the condition of low volume fractions. It is necessary to make sure that δ*_i_* does not exceed 0.5.

In this particular case, we simply regard the pure GAC with no pore structure, namely the “the ideal pure GAC”, as the host medium. Its complex permittivity is characterized as $\varepsilon_{GAC}^{t}$. The value of $\varepsilon_{GAC}^{t}$ should only exist in theory as it can not be measured directly. Similarly, we regard the pore as the inclusions. Thus, the whole GAC is composed of pure GAC and pores. The complex permittivity (ε*_eff_*) of the GAC should be the mixture.

With several steps of elementary algebraic transformation, the MG effective permittivity represented by Equation (1) can be solved by:

(2) $$\varepsilon_{eff}=\varepsilon_{GAC}^{t}\frac{2\delta_{i}\left(\varepsilon_{i}-\varepsilon_{GAC}^{t}\right)+\varepsilon_{i}+2\varepsilon_{GAC}^{t}}{2\varepsilon_{GAC}^{t}+\varepsilon_{i}+\delta_{i}\left(\varepsilon_{GAC}^{t}-\varepsilon_{i}\right)}$$

We simply assume the pore is full of air, which means ε*_i_* = 1. Taking the defining equation of δ*_i_* into account, we obtain:

(3) $$\left\{ \begin{array}{l} \varepsilon_{eff}=\varepsilon_{GAC}^{t}\frac{1+2\delta_{i}+2\left(1-\delta_{i}\right)\varepsilon_{GAC}^{t}}{1-\delta_{i}+\left(2+\delta_{i}\right)\varepsilon_{GAC}^{t}}\\ \delta_{i}=\frac{V_{i}}{V_{total}},\;\left(0<\delta_{i}<0.5\right) \end{array}\right.$$

where *V_i_* is the volume of the inclusions and *V*_total_ is the total volume of the whole GAC. Particularly, when it is for the unit mass of the GAC, *V_i_* turns to be a significant physical concept–the pore volume (*v_g_*) in cm^3^/g.

We introduce the granule density (ρ*_g_*), which is defined as the mass (*m*_0_) divided by the volume (*v*_0_) in a single granule, i.e., ρ*_g_ = m*_0_*/v*_0_. This approach offers two ways to build our model to make the connection between ε*_eff_* and *v_g_*.

### 2.1. Model A

**Figure 1:**
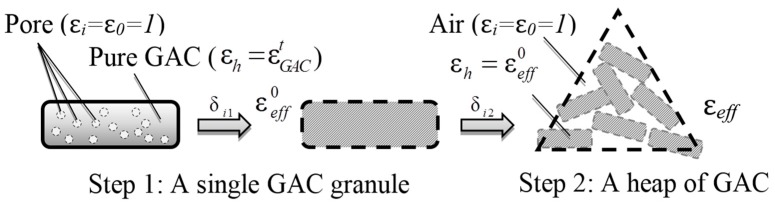
Schematic diagram for deriving the relationship between ε*_eff_* and *v_g_*.

Step by step with applying the MGA Equation (3) twice. As is shown below (Figure 1), it takes two steps to make the connection between ε*_eff_* and *v_g_*.

Step 1: Focusing on a single GAC granule.

Volume fraction δ*_i_* of a single granule should be expressed as follows:

(4) $$\delta_{i}=\frac{V_{i}}{V_{total}}=\frac{v_{g}m_{0}}{v_{0}}=v_{g}\rho_{g}$$

Substituting this relationship into Equation (3):

(5) $$\varepsilon_{eff}^{0}=\varepsilon_{GAC}^{t}\frac{1+2v_{g}\rho_{g}+2\varepsilon_{GAC}^{t}-2v_{g}\rho_{g}\varepsilon_{GAC}^{t}}{1-v_{g}\rho_{g}+2\varepsilon_{GAC}^{t}+v_{g}\rho_{g}\varepsilon_{GAC}^{t}}$$

Step 2: Considering a heap of GAC.

Whereby the volume fraction is calculated as follows:

(6) $$\delta_{i}=\frac{V_{i}}{V_{total}}=\frac{V_{heap}-V_{host}}{V_{heap}}=\frac{V-M/\rho_{g}}{V}=1-\frac{M}{V\rho_{g}}$$

where *M* and *V* are the mass and the volume of the heap, respectively, and *V_host_* is the volume of the host media (i.e., the total volume of the GAC granule in the heap).

Similarly, substituting Equation (6) into (3):

(7) $$\varepsilon_{eff}=\varepsilon_{eff}^{0}\frac{3-\frac{2M}{V\rho_{g}}+\frac{2M}{V\rho_{g}}\varepsilon_{eff}^{0}}{\frac{M}{V\rho_{g}}+3\varepsilon_{eff}^{0}-\frac{M}{V\rho_{g}}\varepsilon_{eff}^{0}}$$

Equation (7) is simplified by multiplying the numerator and denominator with *V∙*ρ*_g_*:

(8) $$\varepsilon_{eff}=\varepsilon_{eff}^{0}\frac{3V\rho_{g}-2M+2M\varepsilon_{eff}^{0}}{M+3V\rho_{g}\varepsilon_{eff}^{0}-M\varepsilon_{eff}^{0}}$$

where *M*, *V*, ρ*_g_*, and $\varepsilon_{GAC}^{t}$ are constant. *M*, *V*, and ρ*_g_* can be measured directly and conveniently. The independent variable *v_g_* is implied in the expression of $\varepsilon_{eff}^{0}$ in Equation (5).

### 2.2. Model B

Directly focus on a heap of GAC, applying MGA Equation (3) only once. In this particularly situation, we simply treat porosity both in the GAC granule (i.e., total pore volume) and between each GAC granules the same. They are all regarded as inclusions, sharing the same permittivity: ε*_i_* = ε_0_ = 1. In this instance, the volume fraction will be less complicated, which is expressed as follows:

(9) $$\delta_{i}=\frac{V_{i}}{V_{total}}=\frac{V_{pores}+V_{air}}{V_{heap}}=\frac{Mv_{g}+\left(V-M/\rho_{g}\right)}{V}=1+\left(v_{g}-\frac{1}{\rho_{g}}\right)\frac{M}{V}$$

Substituting Equations (9) into (3), yielding:

(10) $$\varepsilon_{eff}=\varepsilon_{GAC}^{t}\frac{3+\frac{2M}{V}\left(v_{g}-1/\rho_{g}\right)-\frac{2M}{V}\left(v_{g}-1/\rho_{g}\right)\varepsilon_{GAC}^{t}}{-\frac{M}{V}\left(v_{g}-1/\rho_{g}\right)+\left[3+\frac{M}{V}\left(v_{g}-1/\rho_{g}\right)\right]\varepsilon_{GAC}^{t}}$$

Multiply the numerator and denominator with *V∙*ρ*_g_*, and simplify it, finally we obtain:

(11) $$\varepsilon_{eff}=\varepsilon_{GAC}^{t}\frac{3V\rho_{g}+2M\left(v_{g}\rho_{g}-1\right)-2M\left(v_{g}\rho_{g}-1\right)\varepsilon_{GAC}^{t}}{3V\rho_{g}\varepsilon_{GAC}^{t}-M\left(v_{g}\rho_{g}-1\right)+M\left(v_{g}\rho_{g}-1\right)\varepsilon_{GAC}^{t}}$$

Similarly, *M*, *V*, ρ*_g_*, and $\varepsilon_{GAC}^{t}$ are constant or measurable, it is obvious that the Equation (11) achieved the goal to make the connection between ε*_eff_* and *v_g_*.

## 3. Experiment Setup

### 3.1. Materials and Sample Preparations

The original GAC came from the commercial activated carbon (Analytical pure, KELON Chemical Reagent Factory, Chengdu, China). All of the GAC samples are dried for 2 days at 90 °C with the electric drying oven, except sample B, which was initially soaked in deionized water and then be frozen at −40 °C for 1 day. This process was done to physically enlarge the pore volume. The other samples were named as A, C1, C2 and C3. Generally, sample A was the blank, sample B was treated by freezing procedure, samples C1–C3 were treated by microwave irradiation with a modified domestic microwave oven. The modification involved replacing the magnetron by an industrial one, which is cooled by water and the model named as 2M410A. All of the samples C1–C3 were treated by a microwave in the same anode current 0.4 A, but for different durations (min). In addition, to prevent the GAC from over treatment and potentially burning down, the maximum microwave irradiation duration should be less than 3 min. Thus, in order to observe the effect of different microwave irradiation duration on the properties of GAC samples, the microwave irradiation duration for samples C1–C3 was set to be 1, 2, and 3 min, respectively.

### 3.2. Measurement and Characterization

(a) Measurement of the effective complex permittivity: resonant cavity method.

Specific measurement apparatus and particular principles were referenced in [17]. In general, the measurement system was composed of two parts:

- A metal can cavity and the accompanying open-end coaxial probe were well-designed. The can was finely manufactured with a fixed size. Thus, when the can was filled with the GAC samples, the heap volume V was the same as the volume of the can.
- A vector network analyzer (VNA) (N5230A, Agilent, Santa Rosa, United States) was used to measure the magnitude and phase of scattering parameter S11. Based on a well-trained back propagation (BP) neural network as the core algorithm, it was quite convenient to infer the effective complex permittivity of the sample.

(b) Measurement of the fundamental physical coefficient: the heap mass M, and the granule density ρ*_g_*.

A total of 20–30 grains of GAC were picked arbitrarily. The mass and geometric dimensions of each granular was determined by electronic balance and vernier caliper, respectively. The data was used to calculate the arithmetic mean of the mass and volume for each grain. Finally, the mass of the granular was divided by the volume to obtain the density ρ*_g_*.

(c) Characterization of the pore volume.

Pore volume was determined using the automatic surface area and pore analyzer (ASAP2020, Micromeritcs, Norcross, GA, USA). Nitrogen isotherm adsorption (at relative pressure of 0.99 atm) was used to calculate the pore volume by the static volumetric method.

## 4. Results and Discussion

The detailed experimental results are collected in Table 1.

In Table 1, compared the properties of sample A to sample B, it showed that the frozen process can enlarge the pore volume by about 13.8% or (0.4801 − 0.4219)/0.4219 and reduce the real and imaginary part of the complex permittivity by about 9.3% or (6.419 − 5.824)/6.419 and 13.0% or (1.401 − 1.219)/1.401, respectively. Similarly, the pore volume of samples C1–C3 was enlarged by the microwave irradiation, while the permittivity decreased accordingly. However, we can also noticed that when the microwave radiation duration reaches a certain extent, the pore volume of the sample will be decreased, as shown for sample C3 in Table 1. Therefore, in order to prevent the sample from over treatment and potentially burning down, a reasonable microwave radiation duration was required.

In order to further study the relationship between the pore volume and the permittivity of the GAC samples, we take the average density ($\bar{\rho_{g}}$ = 0.7673 g/cm^3^) and heap mass ($\bar{M}$ = 409.483 g) to represent the GAC’s granule density and heap mass, respectively. Substituting $\bar{\rho_{g}}$, $\bar{M}$, the heap volume (*V* = 785.398 cm^3^) and the measured pore volume (*v_g_*) of each GAC in Table 1 into Equations (5), (8), and (11), we can obtain the $\varepsilon_{GAC}^{t}$ in both Model A and Model B. The results are presented in Table 2 and Table 3.

From Table 2 and Table 3, we can clearly see that the results obtained by Model A are slightly better than those obtained by Model B. The maximum difference of the real and imaginary part of complex permittivity and the loss tangent $\tan\delta \left(\varepsilon_{GAC}^{t}\right)$ obtained by model A and model B is 4.930% or (17.750 − 16.875)/17.750, 7.100% or (2.000 − 1.856)/2.000, and 2.496% or (0.1963 − 0.1914)/0.1963, respectively. Nevertheless, under the condition that there are individual differences in the samples, Table 2 and Table 3 verified that model A and B could provide the results with little difference. Hence, in the following discussion, we just take the data of Model A into account. Considering that the loss tangent plays a crucial role in microwave heating, the results can be clearly grouped according to whether it was processed with microwave. To illustrate this more specifically, we take the average of the non-microwave treatment samples A and B:

(12) $$\varepsilon_{GAC}^{t}=\bar{\varepsilon_{GAC}^{t}}=15.269-j3.766$$

**Figure 2:**
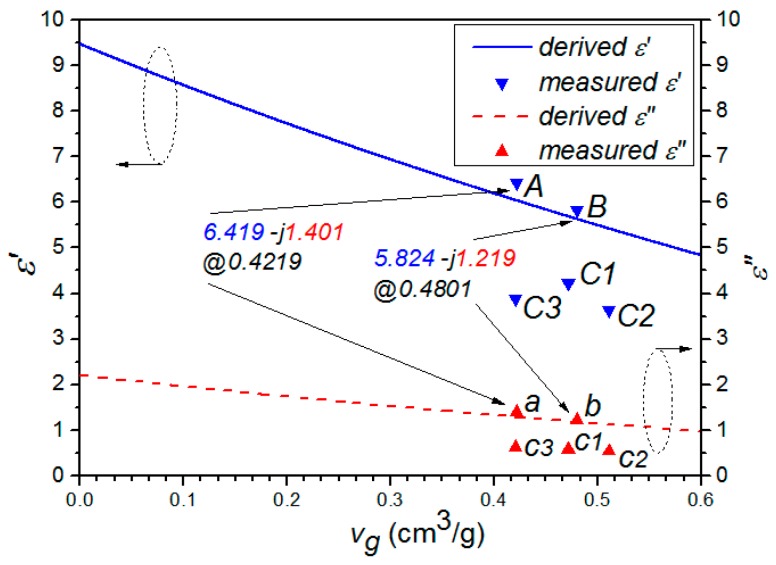
Theoretical and experimental comparison graph between ε*_eff_* (ε′-jε″) and *v_g_*.

**Figure 3:**
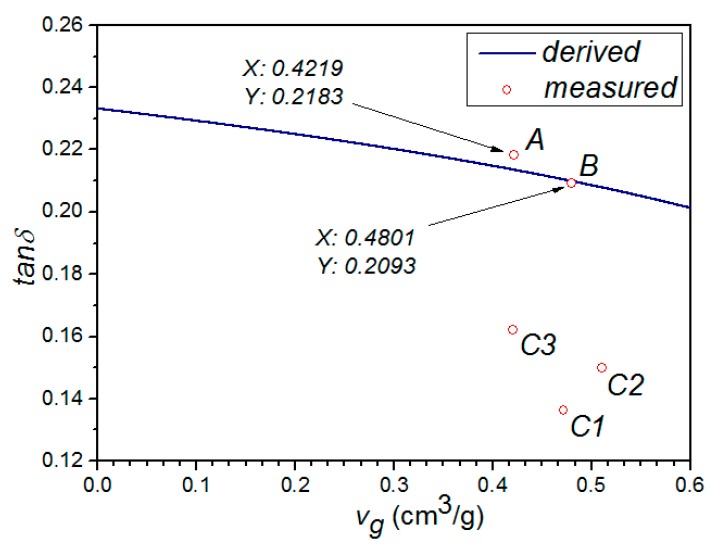
Theoretical and experimental comparison graph between tan δ(ε*_eff_*) and *v_g_*.

Substituting $\bar{\rho_{g}}$, $\bar{M}$, *V*, and Equations (12) into (8), we can derive the relationship between ε*_eff_* and *v_g_*, as shown in Figure 2. Similarly, the relationship between tan δ(ε*_eff_*) and *v_g_* can also be obtained, as shown in Figure 3.

In Figure 2 and Figure 3, it can be clearly seen that the properties of samples A and B fit the derived curve well, while those of samples C1–C3 have a considerable shift from the curve. It indicated that microwave treatment have a significant effect on the structure of GAC, making the classical model no longer suitable for describing its permittivity. This is reasonable, because the derivation in both Model A and Model B did not take the microwave factor into account. Meanwhile, we can also notice that both ε*_eff_* and tan δ(ε*_eff_*) are decreased with the increment of *v_g_*. This may be due to the fact that the air (low permittivity) composition in mixture increases with the increment of *v_g_*.

## 5. Conclusions

This paper studied the relationship between the effective complex permittivity and pore volume of GAC. The relationship was derived based on MGA theory. Two quantificational expressions were established in different equivalent models, respectively. A well-designed resonant cavity and the accompanying open-end coaxial probe were introduced to measure the effective complex permittivity of the GAC. The automatic surface area and pore analyzer was also applied to carry out the BET testing to acquire the pore volume of the GAC. Results obtained by the two expressions were extremely similar. Meanwhile, the theoretical results are in a good agreement with those from the experiment. In addition, the results indicated that microwave treatment had a great impact on the structure of GAC, making the classical model no longer suitable for describing its permittivity.

This study provided a bridge to link the effective complex permittivity and pore volume of GAC. In addition, it provided a potential and convenient method for the rapid assisted characterization of GAC. In the future study, we will try to take the microwave factor into account during derivation, and study the relationship between the permittivity and pore size distribution or different absorbability (porosity).

## Figures and Tables

**Table 1:** The detailed experimental data.

Marker	Treatment	ρ*_g_* (g/cm^3^)	*M* (g)	*V* (cm^3^)	ε*_eff_*	*v_g_* (cm^3^/g)
A	—	0.8214	424.757	785.398	6.419-j1.401	0.4219
B	Frozen	0.7072	431.935		5.824-j1.219	0.4801
C1	Microwave Irradiation	0.7791	404.441		4.233-j0.577	0.4718
C2		0.7683	379.004		3.630-j0.544	0.5108
C3		0.7601	407.279		3.876-j0.629	0.4206
Average		0.7673	409.483		–	0.46104

**Table 2:** The complex permittivity of the ideal perfect granular activated carbon (GAC) in Model A.

Marker	Treatment	$\varepsilon_{GAC}^{t}$	$\tan\delta \left(\varepsilon_{GAC}^{t}\right)$
A	—	17.750-j4.460	0.2513
B	Frozen	12.789-j3.071	0.2402
C1	Microwave Irradiation	11.169-j1.896	0.1697
C2		10.191-j2.000	0.1963
C3		8.883-j1.804	0.2031

**Table 3:** The complex permittivity of the ideal perfect GAC in Model B.

Marker	Treatment	$\varepsilon_{GAC}^{t}$	$\tan\delta \left(\varepsilon_{GAC}^{t}\right)$
A	—	16.875-j4.180	0.2477
B	Frozen	12.411-j2.945	0.2373
C1	Microwave Irradiation	10.683-j1.776	0.1663
C2		9.708-j1.858	0.1914
C3		8.590-j1.711	0.1992
